# Prognostic Value of Plasma hPG_80_ (Circulating Progastrin) in Metastatic Renal Cell Carcinoma

**DOI:** 10.3390/cancers13030375

**Published:** 2021-01-20

**Authors:** Manish Kohli, Winston Tan, Bérengère Vire, Pierre Liaud, Mélina Blairvacq, Frederic Berthier, Daniel Rouison, George Garnier, Léa Payen, Thierry Cousin, Dominique Joubert, Alexandre Prieur

**Affiliations:** 1 Division of Oncology, Department of Medicine, Huntsman Cancer Institute, 2000 Circle of Hope Dr., Salt Lake City, UT 84112, USA; 2 Division of Hematology-Oncology, Mayo Clinic, Jacksonville, FL 32224, USA; tan.winston@mayo.edu; 3 Eurobiodev, 2040 Avenue du Père Soulas, 34000 Montpellier, France; b.vire@eurobiodev.com (B.V.); p.liaud@eurobiodev.com (P.L.); m.blairvacq@eurobiodev.com (M.B.); 4 Centre Hospitalier Princesse Grace, 1 Avenue Pasteur, Principauté de Monaco, 98000 Monaco, Monaco; frederic.berthier@chpg.mc (F.B.); daniel.rouison@chpg.mc (D.R.); georges.garnier@chpg.mc (G.G.); 5 Laboratoire de Biochimie et Biologie Moleculaire, CITOHL, Centre Hospitalier Lyon-Sud, 69310 Pierre-Bénite, France; lea.payen-gay@chu-lyon.fr; 6 ECS-Progastrin, Chemin de la Meunière 12, 1008 Prilly, Switzerland; t.cousin@ecs-progastrin.com (T.C.); d.joubert@ecs-progastrin.com (D.J.)

**Keywords:** metastatic renal cell carcinoma (mRCC), hPG_80_, IMDC, blood-based prognostic biomarker, circulating progastrin

## Abstract

**Simple Summary:**

Metastatic renal cell carcinoma (mRCC) accounts for one-third of all newly diagnosed renal cell cancers. A better understanding of the biology and molecular basis of disease progression has resulted in several drug targets being identified and led to approval of several new drugs for treating mRCC in the past decade. A growing need has emerged for identifying novel molecular tumor biology based and stage-specific prognostic and predictive biomarkers in mRCC reflective of biology beyond the currently available prognostic models which are solely based on clinical characteristics. We investigated hPG80 (circulating progastrin), which is associated with kidney cancer biology and found that hPG80 levels is both an independent prognostic marker in mRCC and also improves current clinical prognostic models. This will help stratify mRCC patients more accurately in future and improve the management of mRCC patients.

**Abstract:**

Precise management of kidney cancer requires the identification of prognostic factors. hPG_80_ (circulating progastrin) is a tumor promoting peptide present in the blood of patients with various cancers, including renal cell carcinoma (RCC). In this study, we evaluated the prognostic value of plasma hPG_80_ in 143 prospectively collected patients with metastatic RCC (mRCC). The prognostic impact of hPG_80_ levels on overall survival (OS) in mRCC patients after controlling for hPG_80_ levels in non-cancer age matched controls was determined and compared to the International Metastatic Database Consortium (IMDC) risk model (good, intermediate, poor). ROC curves were used to evaluate the diagnostic accuracy of hPG_80_ using the area under the curve (AUC). Our results showed that plasma hPG_80_ was detected in 94% of mRCC patients. hPG_80_ levels displayed high predictive accuracy with an AUC of 0.93 and 0.84 when compared to 18–25 year old controls and 50–80 year old controls, respectively. mRCC patients with high hPG_80_ levels (>4.5 pM) had significantly lower OS compared to patients with low hPG_80_ levels (<4.5 pM) (12 versus 31.2 months, respectively; *p* = 0.0031). Adding hPG_80_ levels (score of 1 for patients having hPG_80_ levels > 4.5 pM) to the six variables of the IMDC risk model showed a greater and significant difference in OS between the newly defined good-, intermediate- and poor-risk groups (*p* = 0.0003 compared to *p* = 0.0076). Finally, when patients with IMDC intermediate-risk group were further divided into two groups based on hPG_80_ levels within these subgroups, increased OS were observed in patients with low hPG_80_ levels (<4.5 pM). In conclusion, our data suggest that hPG_80_ could be used for prognosticating survival in mRCC alone or integrated to the IMDC score (by adding a variable to the IMDC score or by substratifying the IMDC risk groups), be a prognostic biomarker in mRCC patients.

## 1. Introduction

Metastatic renal cell carcinoma (mRCC) accounts for one-third of 73,750 new RCC patients at initial presentation in the US [1] and 14,380 deaths are estimated in 2020 [2]. mRCC is a group of heterogeneous tumor types characterized by distinct biologic patterns and prognosis [3,4]. In the past 15 years, the introduction of targeted therapy, and more recently immunotherapy, have increased the therapeutic armamentarium available to treat metastatic stage and increased overall survival (OS) [5,6,7]. In parallel, major technological advances have led to a better understanding of the molecular basis of disease progression and the identification of several prognostic biomarkers [8,9,10]. However, well-validated prognostic biomarkers associated with tumor biology of metastatic kidney cancer stage are lacking.

Recent clinical trials have used the International Metastatic Database Consortium (IMDC) model for prognostication [11,12,13]. This model is based on six clinical variables as risk factors for short survival (time from diagnosis to initiation of therapy of less than 1 year, Karnofsky performance status of less than 80%, serum hemoglobin below the lower limit of normal and corrected calcium, neutrophil count, and platelet count greater than the upper limit of normal) and classify patients into good- (0 risk factor), intermediate- (1 or 2 risk factors) and poor-risk (≥3 risk factors) groups [14]. As a result, targeted therapeutics in metastatic RCC uses non-molecular based clinical prognostic variables for initiating novel drug interventions and combinations highlighting the need for enhancing prognostic markers in metastatic RCC [15,16].

We have reported previously [17] progastrin to be elevated in plasma of patients with metastatic kidney cancer. In physiology, progastrin is the precursor of gastrin synthetized by antrum G cells and processed into gastrin [18]. Progastrin does not accumulate in G cells, by contrast to G34-Gly and gastrin [19]. G34-Gly will generate gastrin upon full maturation. As a consequence, progastrin is barely detectable in the blood of healthy subjects even though few of them have been shown to be positive as observed the first time by Siddheshwar et al. [20]. However, in line with the demonstration of the expression of the *GAST* gene, encoding progastrin, in colorectal tumors as well as other tumor types, high levels of hPG_80_ (named as such when progastrin is released from tumor cells and detected in the blood) were reported in the blood of cancer patients [17,20,21]. Moreover, in addition to the fact that *GAST* is a direct target of the ß-catenin/Tcf4 pathway, activated in many cancers, including RCC [22,23], a large body of literature supports the functional role of hPG_80_ in tumorigenesis [21,24,25,26,27,28]. As a consequence, hPG_80_ is an interesting indicator of tumor behavior/activity and may also represent a factor for aggressive biology and clinical outcomes.

In the present study, we determined the prognostic value of hPG_80_ in clinically diagnosed metastatic RCC (mRCC) patients and examined whether hPG_80_ might improve stratification of the currently used IMDC model to predict overall survival (OS).

## 2. Materials and Methods

### 2.1. Patients and Control Cohorts

**Figure 1:**
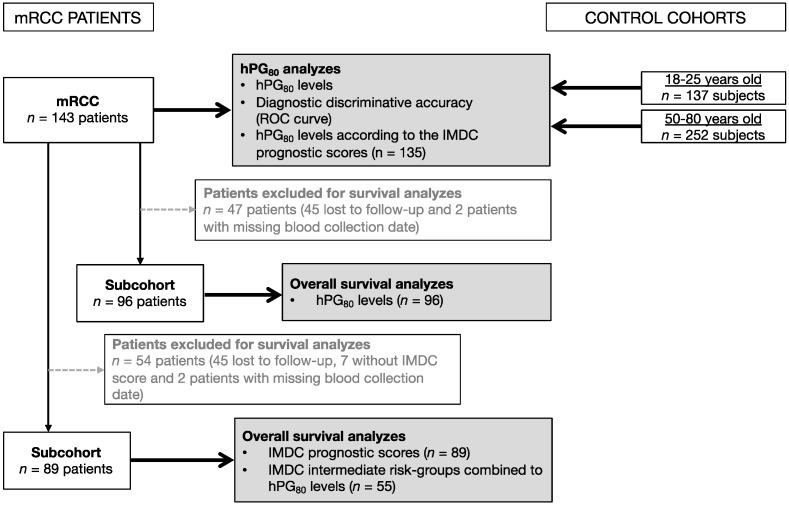
Flowchart of the study with mRCC patients.

Figure 1 illustrates the different cohorts used in this study to determine prognostic value of hPG_80_ in metastatic RCC stage.

#### 2.1.1. mRCC Patient Cohort

A large tertiary level, clinically annotated hospital registry with prospective and uniform blood/plasma collection from non-fasting metastatic kidney cancer patients between 5/2011 and 9/2013 and uniform sampling was used as has been previously described [29,30]. All patients (*n* = 143) provided written informed consent for research at the time of their blood collection, in line with international regulations and ICH GCP (International Conference on Harmonization- Good Clinical Practice) and on an Institutional Review Board approved study protocol (Mayo Clinic IRB #11-005855 00). Of note, the majority of the patients had the blood draw close to the time of the diagnosis of metastatic disease, with a median value of 6 days (IQR: 0–117).

#### 2.1.2. RCC Patient Cohort

Blood samples from non-fasting RCC patients (*n* = 39) were obtained from Tissue For Research Ltd. (Spectrum Health System, Grand Rapids, Michigan, MI, USA).

#### 2.1.3. Non-Cancer Age and Non Age Matched Control Cohorts

A young-aged cohort of plasma samples (18–25 years old) was obtained from non-fasting 137 healthy blood donors, from the French blood agency (*Etablissement Français du Sang*). This cohort was assumed to be at very low risk of cancer in order to maximize the likelihood of the control group being cancer-free [31]. A second, over 50 years old cohort of plasma samples (range 50–80 year old; median value 55 year old) was collected from fasting 252 healthy subjects, from the interim analysis from the PROCODE study (NCT03775473, https://clinicaltrials.gov/ct2/show/NCT03775473).

### 2.2. hPG_80_ Level Measurements in the Blood Samples

The DxPG_80_ test is an Enzyme-Linked Immunosorbent Assay (ELISA) for the quantitative measurement of human hPG_80_ in EDTA plasma. The test is based on the principle of a sandwich ELISA to measure the concentration of hPG_80_ in plasma specimens that have been anticoagulated with EDTA. Briefly, a capture monoclonal antibody raised against the C-Terminus of hPG_80_ and that does not recognize active gastrin (gastrin-NH2) is pre-coated on the 96-well plate. hPG_80_ present in calibrators, controls and/or specimens added to the wells bind to the immobilized capture antibody. The test plate includes calibrators which are used to estimate the level of hPG_80_ in EDTA plasma samples. The wells are washed and a polyclonal antibody raised against the N-Terminus of hPG_80_ coupled with horseradish peroxidase (HRP) is added (i.e., detection antibody), resulting in an antibody-antigen-antibody complex. After a second wash, a 3,3′,5,5′-Tetramethylbenzidine (TMB) substrate solution is added to the well, producing a blue color in direct proportion to the amount of hPG_80_ present in the initial sample. The Stop Solution changes the color from blue to yellow, and the wells are read at 450 nm with a microplate reader.

The analytical performances were assessed following EMEA/CHMP/EWP/192217/2009. Accordingly, there is no cross-reactivity or interference if the variation in the percentage of recovery (when compared to control (vehicle)) is equal or does not exceed 20%, and there is no change in the interpretation of the result.

Cross-reactivity was assessed using human plasma samples spiked with native hPG_80_ concentrations ranging from negative to strong positive and a fixed concentration of each potential cross-reactants (i.e., KLH (Keyhole Limpet Hemocyanin), gastrin-17, Gastrin-Gly, CTFP (C-Terminus Flanking Peptide) at 2 µg/mL, CA125 (cancer antigen 125) at 2000 U/mL, CA15-3 (cancer antigen 15-3) at 100 U/mL, CEA (carcinoembryonic antigen) at 20 µg/mL or PSA (prostate specific antigen) at 210 mg/mL). For all the potential cross-reactants, the variation of the % of recovery was below 20%, showing no cross-reactivity.

Interference was also assessed using human plasma samples spiked with native hPG_80_ concentrations ranging from negative to strong positive and a fixed concentration of each potentially interfering substances (i.e., SN-38 at 60 µM, 5-FU at 3 mM, triglycerides at 0.05 mg/mL, cholesterol at 25 µg/mL, conjugated bilirubin at 0.5 µg/mL or hemoglobin at 2 mg/mL). For all the potential interfering substances, the variation of the % of recovery was below 20%, showing no interference.

Based on EMEA/CHMP/EWP/192217/2009, the within-run, inter-run and inter-operator variability is defined as being the mean coefficient of variation (CV) % value of each measured control sample. It is considered acceptable when ≤20%. The CV% were determined using hPG_80_-negative EDTA plasma spiked with three controls of hPG_80_ at 2.5, 12.5 and 22.5 pmol/L. For all the variabilities, the CV% was below 10%, showing acceptable variabilities.

hPG_80_ concentrations in pmol/L (pM) were calculated using the standard curve equation of the native hPG_80_ calibrators prepared in hPG_80_-negative EDTA plasmas. The limit of detection (LoD) and limit of quantification (LoQ) were calculated, as per EMEA/CHMP/EWP/192217/2009 and NCCLS EP 17-A vol.24 no.34, based on the standard deviation (CV) of the measured concentrations of *n* = 74 blanks as LoD = 3 × CV and LoQ = 10 × CV. DxPG_80_ have a LoD of 1.2 pM and a LoQ of 2.3 pM.

### 2.3. Statistical Analyses

Comparisons between groups were performed using two-tailed Mann-Whitney *U*-test for unpaired non-parametric variables. Spearman’s rank correlation coefficient was used to measure the association between between hPG_80_ levels and age. The overall survival (OS) was defined as the time from blood collection from mRCC patients with known date of death. The survival curves were constructed using the Kaplan-Meier method and compared performing a log-rank test on mRCC patients with full survival data. An optimal cutoff value of hPG_80_ was defined using the function of “surv_cutpoint” in R Package “survminer”, calculating the minimal *p*-value based on the log-rank method. R software 3.6.1 (The R Foundation for Statistical Computing) was used to perform all the statistical analyses. Prism software (GraphPad, La Jolla, CA, USA) was used to create figures. The level of significance was set at *p* < 0.05.

## 3. Results

### 3.1. Characteristics of the Study Population

**Figure 2:**
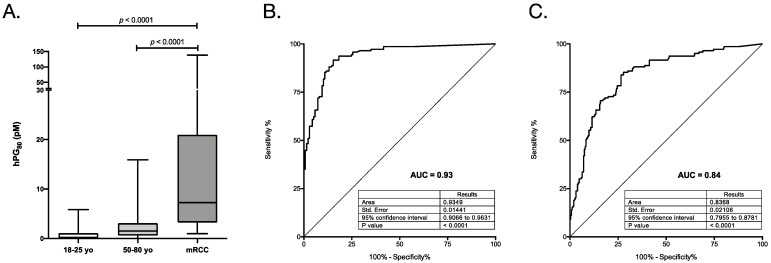
Levels and performances of plasma hPG_80_ in patients with mRCC patients. (**A**) hPG_80_ concentrations in mRCC patients (*n* = 143) compared to 18–25 (*n* = 137) and 50–80 (*n* = 252) years old control groups. Two-tailed Mann-Whitney test. B to C. Diagnostic discriminative accuracies of hPG_80_ in mRCC patients compared to control groups aged 18–25 year old (**B**) or 50–80 year old (**C**) using Receiver Operating Characteristics (ROC) curve analysis.

The demographic characteristics of the mRCC patients and control cohorts (143 mRCC patients to assess the diagnostic and prognostic values of hPG_80_) are shown in Table 1. A flowchart describing the selection of the patients for the study analysis is shown in Figure 1. The median patient age is 63 years and 74.8% were male (Table 1). The majority of the patients had metastatic clear cell renal carcinoma (77%). The median level in the mRCC cohort of plasma hPG_80_ is 7.23 pM (Figure 2A and Table 2). The diagnostic accuracy of hPG_80_ were evaluated in mRCC patients. While the mRCC cohort of 143 patients was divided into IMDC prognostic categories to evaluate hPG_80_ as a prognostic factor in mRCC stage, we performed survival analysis using OS as the clinical outcome only in patients who had survival data available. For survival analysis using only hPG_80_ levels, 47 mRCC patients were excluded (45 lost to follow-up and 2 patients with missing blood collection date) (Figure 1). For survival analysis using the IMDC score, 54 mRCC patients were excluded (45 lost to follow-up, 7 without IMDC score and 2 patients with missing blood collection date) (Figure 1). At the time of analysis, 96 and 89 patients had died from disease progression and the median OS was 19.2 and 20.1 months, for each subcohort.

### 3.2. Diagnostic Performance of hPG_80_ in mRCC Patients

To determine whether hPG_80_ plasma levels can be used as a biomarker for mRCC diagnosis, we measured hPG_80_ levels in 143 mRCC patients and in the two control groups. hPG_80_ was detected in 94% of the RCC patients (threshold = 1.2 pM, corresponding to the limit of detection of the DxPG_80_ kit). Median hPG_80_ concentration in the mRCC cohort (median value: 7.23 pM) was significantly higher than that in the 18–25 year old control group (median value below the LoD; *p* < 0.0001) (Figure 2A and Table 2). In the 50–80 year old control group, median hPG_80_ concentration was 1.50 pM and was also significantly lower than in mRCC patients (*p* < 0.0001; Figure 2A and Table 2). Although there is a significant difference in hPG_80_ levels between the two control groups (*p* < 0.0001), in each control group there is no correlation between hPG_80_ levels and age (Spearman r = −0.13; *p* = 0.11 and Spearman r = −0.035; *p* = 0.16 for the 18–25 year old and 50–80 year old cohorts respectively).

Receiver operating characteristic (ROC) curves were used to evaluate the diagnostic discriminative accuracy of hPG_80_ levels in mRCC patients compared to the two control groups. hPG_80_ levels displayed high predictive significance, with an area under the curve (AUC) value of 0.93 (95% CI: 0.91–0.96; *p* < 0.0001) when compared to 18–25 year old control group, and of 0.84 (95% CI: 0.80–0.88; *p* < 0.0001) when compared to 50–80 year old control group (Figure 2B,C). To further explore the diagnostic accuracy of hPG_80_, we used a cohort of 39 non-metastatic RCC patients (Figure S1). The demographic characteristics of the RCC patients are shown in Table S1. Consitent with the results obtained with mRCC patients, hPG_80_ was detected in 85% of RCC patients, median hPG_80_ levels was 7.36 pM (IQR: 3.05–18.23) (Figure S2 and Table S2). The diagnostic discriminative accuracy was 0.89 (95% CI: 0.82–0.95; *p* < 0.0001) when compared to the 18–25 year old control group (Figure S2B), and 0.77 (95% CI: 0.68–0.86; *p* < 0.0001) when compared to the 50–80 year old control group (Figure S2C).

### 3.3. OS for Patients with mRCC Stratified Using hPG_80_ Levels

**Figure 3:**
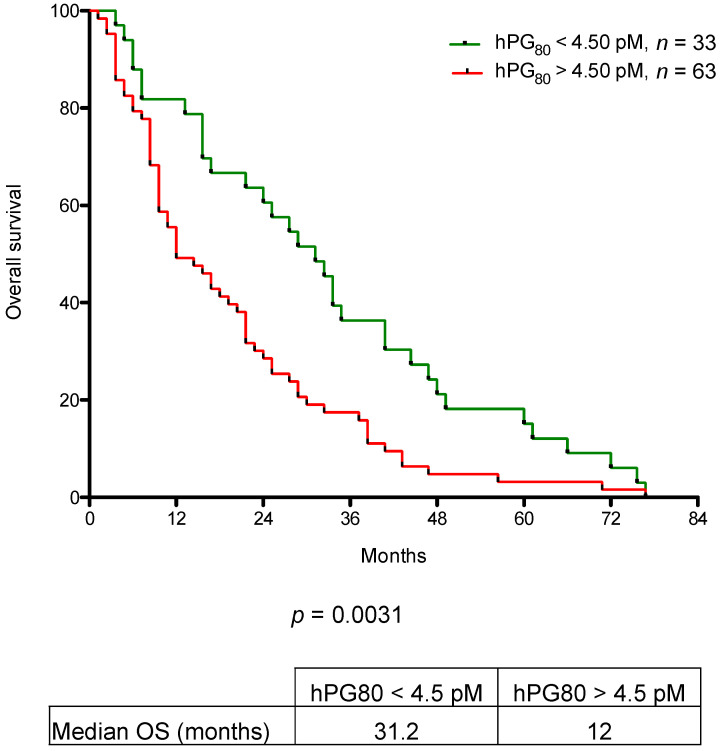
Overall survival according to hPG_80_ levels. Kaplan-Meier analysis of OS for mRCC patients according to hPG_80_ levels. The *p* values from the log-rank test are indicated.

We first investigated the prognostic value of hPG_80_ level in the absence of any other clinical parameters by stratification of patients into high and low hPG_80_ levels based on an optimal calculated cutoff value of hPG_80_ levels across all mRCC patients. The optimal cutoff value of hPG_80_ was obtained using the function of “surv_cutpoint” in R Package “survminer” as described in the Material and Methods section and then used for analysis of survival outcomes. We used 4.5 pM as the optimal cutoff value and patients were segregated into 2 groups above and below this value: 33 patients displayed low hPG_80_ levels (<4.5 pM) and 63 patients displayed high hPG_80_ levels (>4.5 pM). Kaplan-Meier survival analysis showed that patients with high hPG_80_ levels (>4.5 pM) had significantly lower OS compared to patients with low hPG_80_ levels (<4.5 pM) (12 versus 31.2 months, respectively; *p* = 0.0031) (Figure 3).

### 3.4. hPG_80_ Levels According to IMDC Prognostic Group

**Figure 4:**
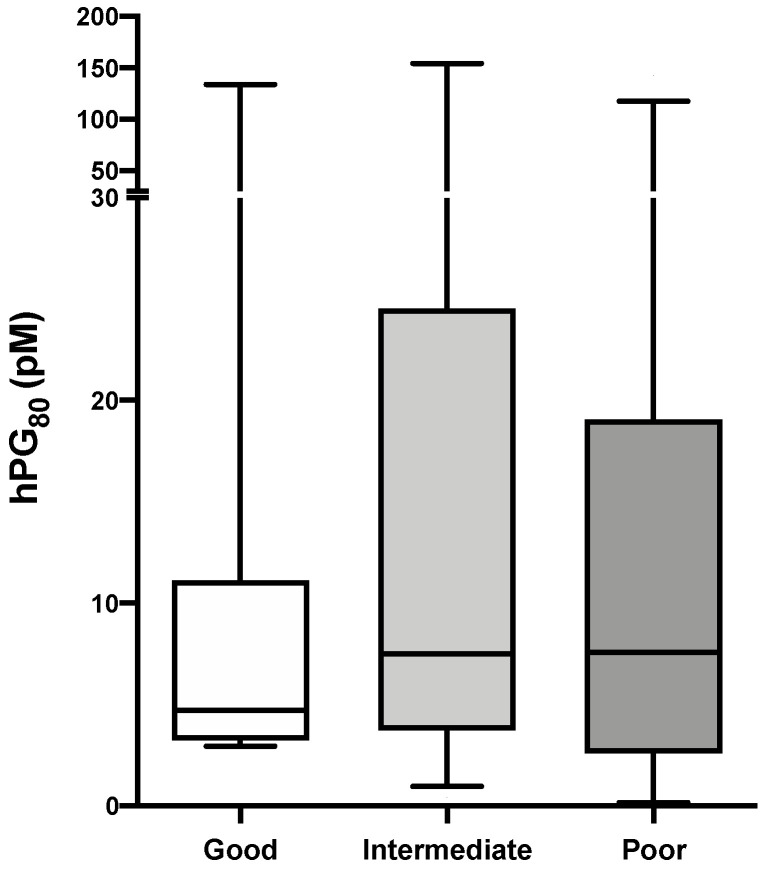
hPG_80_ levels according to the IMDC score. hPG_80_ levels in mRCC patients according to the good-, intermediate- and poor-risk IMDC risk classification. One-tailed Mann-Whitney test; ns: not significant.

Based on the IMDC prognostic criteria, the good, intermediate and poor prognostic categories comprised 28 (19.6%), 81 (56.6%) and 26 (18.2%) patients, respectively (Table 1). We assessed whether the IMDC prognostic score was correlated with hPG_80_ levels. Although the median value for hPG_80_ levels was numerically lower in the good-risk group (4.72 pM) in comparison to the intermediate- and poor-risk groups (7.49 pM, and 7.58 pM, respectively), our results showed that hPG_80_ levels were not statistically different between the IMDC prognostic groups (Figure 4 and Table 3).

### 3.5. OS for Patients with mRCC Stratified Using the IMDC Risk Classification

**Figure 5:**
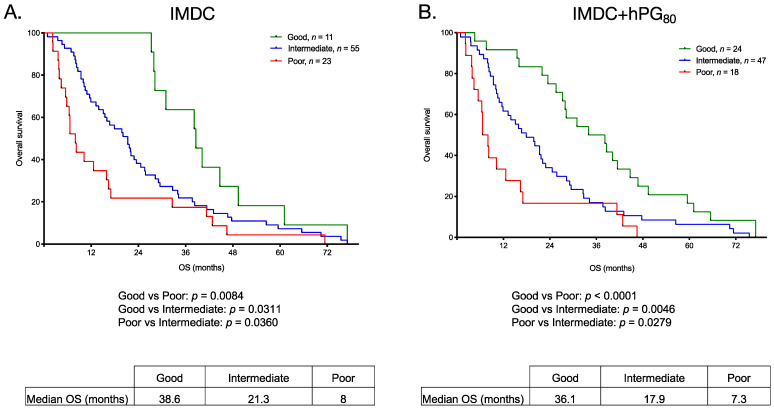
Overall survival according to the IMDC score and to a model incorporating IMDC variables and hPG_80_ levels. (**A**). Kaplan-Meier analysis of overall survival (OS) for mRCC patients according to the IMDC risk classification (good, intermediate, poor). (**B**). Kaplan-Meier analysis of OS for mRCC patients with a model combining IMDC variables and hPG_80_ levels. The median OS (in months) and the *p* values from the log-rank test are indicated.

We evaluated the performance of the IMDC prognostic score and its association with OS in our cohort for all patients with survival data (Flow chart on Figure 1). In this subgroup, the good-, intermediate- and poor-risk groups comprised 11 (12.4%), 55 (61.8%) and 23 (25.8%) patients, respectively. The median OS stratified by IMDC risk classification was 38.6, 21.3 and 8 months for the good-, intermediate- and poor-risk groups, respectively (Figure 5A). A significant difference in OS was observed between the three groups (*p* = 0.0076). The OS for patients in the poor-risk group was significantly shorter compared to patients in the good- and intermediate risk groups (*p* = 0.0084 and *p* = 0.0360, respectively). The OS was also significantly shorter between patients in the intermediate-risk group and the good-risk group (*p* = 0.0311).

### 3.6. OS for Patients with mRCC Stratified Using the IMDC Risk Classification and High and Low hPG_80_ Levels

As hPG_80_ levels represent an independent variable associated with OS in mRCC patients, we assessed whether the IMDC risk classification to predict OS could be improved by incorporating hPG_80_ levels. This model incorporates the six variables from the IMDC risk classification (score from 0 to 6) and hPG_80_ levels (score of 1 for patients having hPG_80_ levels > 4.5 pM and 0 for patients with hPG_80_ levels < 4.5 pM). Patients were categorized using three-level risk groups: good (score = 0–1), intermediate (score = 2–3) and poor (score ≥ 4). Using this model, the good-, intermediate- and poor-risk groups comprised 24 (27%), 47 (52.8%), and 18 (20.2%) patients, respectively. As shown in Figure 5B, the median OS stratified by this new model was 36.1, 17.9 and 7.3 months for the good-, intermediate- and poor-risk groups, respectively. A greater and significant difference in OS was observed between the three groups (*p* = 0.0003 compared to *p* = 0.0076 with the IMDC score). Similarly to what we observed using the IMDC risk classification alone, the OS for patients in the poor-risk group was significantly shorter, compared to patients in the good- and intermediate risk groups (*p* < 0.0001, and *p* = 0.0279, respectively). There was slighty better significance achieved using hPG_80_ levels > 4.5 pM than the clinical variables inthe IMDC risk classification alone while discriminating the three prognostic groups (Figure 5) and more refined discrimination for survival for patients in the intermediate-risk group and the good-risk group (*p* = 0.0046).

### 3.7. OS in The IMDC Intermediate-Risk Group Stratified Using hPG_80_ Levels

**Figure 6:**
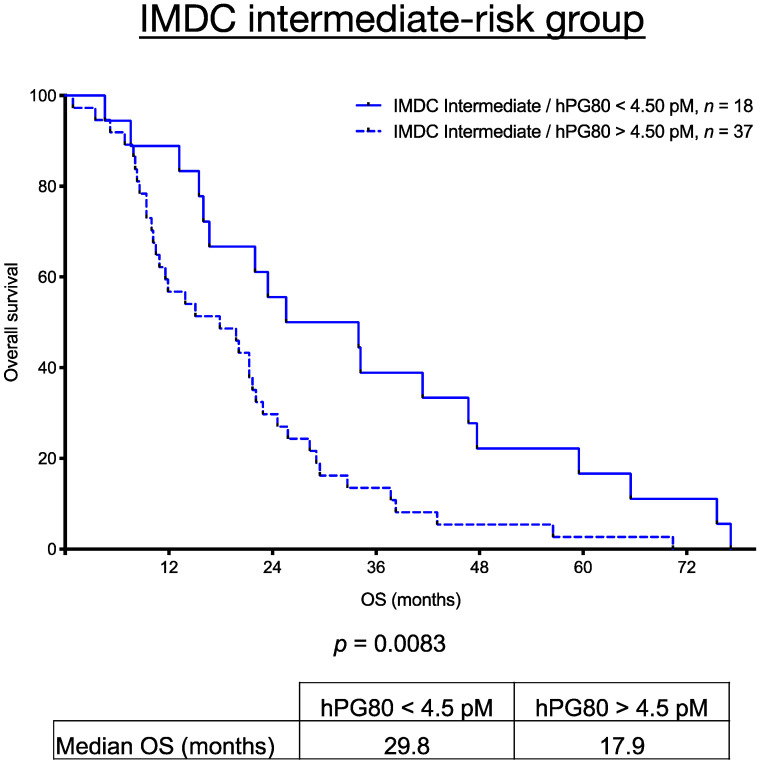
Overall survival in the IMDC intermediate-risk group stratified according to hPG_80_ levels. Kaplan-Meier analysis of OS for mRCC patients according to the IMDC risk classification and incorporating hPG_80_ levels. Patients in the IMDC intermediate-risk group were divided into two groups based on the calculated cutoff value of hPG_80_ (4.5 pM): 18 patients displayed low hPG_80_ levels (<4.5 pM) and 37 patients displayed high hPG_80_ levels (>4.5 pM). The median OS (in months) and the *p* values from the log-rank test are indicated.

Based on the IMDC risk classification, patients in the intermediate-risk group harbor either one or two risk factors [14]. Consequently, there is a great variability and a lack of refinement for survival outcomes using clinical variables alone among these patients. We stratified the intermediate risk group mRCC patients by combining hPG_80_ levels with IMDC risk classification in order to analyse if it could enhance the accuracy of prognostification over IMDC classification alone. We divided the 55 mRCC patients into 2 subgroups based on the optimal calculated cutoff values of 4.5 pM described above for hPG_80_ across the mRCC patients analysed for survival outcomes. As shown in Figure 6, when patients in intermediate IMDC group were stratified into two groups based on hPG_80_ levels cutoff, the OS of patients with hPG_80_ levels > 4.5 pM were significantly lower than the one from the group with hPG_80_ levels < 4.5 pM (median OS: 17.9 versus 29.8 months; *p* = 0.0083).

## 4. Discussion

The close relationship between hPG_80_ and cancer progression has been previously reported in several studies [21,24,25,26,27,28]. We have recently shown that tumor cells from many cell types overexpress hPG_80_ [17]. Its expression is related to cancer cell activity and represents a risk factor for tumor recurrence and therefore hPG_80_ may provide prognostic significance in metastatic stages of tumors which secrete hPG_80_ in measurable amounts. In this study, we demonstrate for the first time the prognostic value of hPG_80_ in mRCC. We also demonstrated the potential diagnostic utility of hPG_80_ levels in differentiating RCC and mRCC patients from healthy individuals. We established an optimal cutoff value for hPG_80_ (4.5 pM) in mRCC stage and were able to demonstrate that higher hPG_80_ levels are associated significantly with lower OS. We also explored hPG_80_ levels in mRCC patients for stratification into good and poor prognosis groups like the widely used IMDC model who classify patients into three risk groups (good, intermediate and poor) [14].

Our results suggest that addition of hPG_80_ levels to IMDC might further improve the IMDC prognostication, and also refines the heterogeneity within the intermediate-risk IMDC group. Patients in the intermediate-risk group account for 40 to 50% of all risk classes patients [32,33]. In our patient cohort, 56.6% of patients had IMDC intermediate-risk group. Study from Sella et al. has shown that patients with one-risk factor had longer OS than patients with two-risk factors, suggesting that adding new prognostic risk factors to actual IMDC risk model such as tumor and stage associated hPG_80_ levels could enhance the accuracy of prognostification [34]. We observed that hPG_80_ levels collected and measured at the time of patients progressing to metastatic stage were able to refine the existing clinical factor based IMDC risk model prognostication of survival in patients with intermediate-risk group. In this group patients with low and high hPG_80_ levels were able to be identified with different survival time (median OS: 17.9 in patients with high hPG_80_ levels versus 29.8 months with low hPG_80_ levels; *p* = 0.0083). In line with these data, a recent study from Kunishi et al. has shown that combining C-reactive protein (CRP) value to patients classified in the intermediate group according to the IMDC risk classification might further refine prognosis in this patient populations [35]. Altogether, although the optimal hPG_80_ cutoff value will need further validation in larger cohort, it supports the benefit of further studies using hPG_80_ levels to stratify mRCC patients and to improve the prognostic predictive power of the existing IMDC model.

Novel tissue and blood-based biomarkers including PD-L1 expression, CRP, VEGF and IL-6 serum levels have been proposed as prognostic biomarkers for mRCC [36]. Other putative biomarkers such as miRNAs, circulating DNA and metabolic biomarkers have been reported to have prognostic relevance in mRCC [36]. In addition, assessment of gene expression signatures has also shown to significantly improve the predictive power of the IMDC model and provided additional prognostic information in mRCC patients [37]. However, most of these factors were derived from retrospective studies and none of them have been validated in larger prospective studies. Currently, no specific molecular marker has been shown to improve the accuracy of existing prognostic scores which are largely based on clinical and not tumor biology associated factors (e.g., IMDC) and their use is not recommended for clinical care [38].

Cancer stem cells (CSCs) have been implicated in tumor initiation, progression, metastasis, multidrug resistance and recurrence [39]. Several lines of evidence indicates that renal CSCs are involved in driving RCC progression and treatment failure [40,41]. Previous studies have shown that hPG_80_ plays a major function in CSCs by regulating their survival and self-renewal ability [21]. Thereby, we can hypothesize that elevated hPG_80_ levels are associated with poor clinical outcome in mRCC patients by promoting CSCs survival and resistance to antiangiogenic drugs like tyrosine kinase inhibitors.

Our study presents some limitations including the use of the prospectively enrolled mRCC cohort who were retrospectively analysed and the number of patients excluded from overall survival analysis for a variety of reasons. A third of our patients were lost to follow-up and this could have biased our results. Nevertheless, hPG_80_ levels in mRCC biology has been well reported and the ability to measure hPG_80_ levels in blood provides a significant advantage for its future exploration as a prognostic or predictive biomarker.

## 5. Conclusions

In conclusion, we showed that elevated hPG_80_ levels is strongly associated with RCC and mRCC and could be a useful prognostic biomarker in these patients. If confirmed in larger datasets with longitudinal follow up in future studies, hPG_80_ could help risk-stratify mRCC patients into different prognostic groups and guide patient management.

## Figures and Tables

**Table 1:** Clinical and pathological characteristics for mRCC patients and control cohorts.

Cohorts		mRCC	Controls	
			18–25 Years Old	50–80 Years Old
		*N* (%)	*N* (%)	*N* (%)
		*n* = 143	*n* = 137	*n* =252
Age, years	Median (range)	63 (41–85)	21 (18–25)	55 (50–80)
Gender	Male	107 (74.8%)	79 (57.7%)	99 (39.3%)
	Female	36 (25.2%)	58 (42.3%)	153 (60.7%)
Histology	Clear cell	110	NA	
	Chromophobe	8		
	Papillary	14		
	Other	11		
Fuhrman Grade (G)	G1	3		
	G2	45		
	G3	45		
	G4	32		
	Not applicable	0		
	Unspecified	18		
Sarcomatoid differentiation	Present	16		
	Absent	78		
	Unspecified	49		
Necrosis	Present	66		
	Absent	77		
IMDC prognostic score	Good	28		
	Intermediate	81		
	Poor	26		
	Unspecified	8		
Clinical stage	I	0		
	II	0		
	III	0		
	IV	143		
	Unspecified	0		
Total lines of systemic therapy	0	24		
	1	55		
	2	30		
	3	19		
	4	8		
	5 or more	7		

NA: non applicable.

**Table 2:** hPG_80_ levels in mRCC patients and in control cohorts. <LoD: hPG_80_ levels were below the limit of detection (LoD).

Characteristics		*N*	hPG_80_	
			Median (IQR), pM	Mean (SE), pM
Patients	mRCC	143	7.23 (3.24–20.61)	24.50 (3.75)
Control cohorts	18–25 years old	137	<LoD	<LoD
	50–80 years old	252	1.5 * (0.0–3.09)	3.82 (0.55)

*: Median hPG_80_ concentration in the 50–80 year old control group is comprised between the LoD and the limit of quantification (LoQ) (i.e., 1.2 pM and 2.3 pM).

**Table 3:** hPG_80_ levels according to the IMDC score in mRCC patients.

Characteristics			hPG_80_	
		*N* (%)	Median (IQR), pM	Mean (SE), pM
IMDC prognostic score	Good	28 (20.7%)	4.72 (3.24–11.14)	20.00 (7.18)
	Intermediate	81 (60.0%)	7.49 (3.73–24.55)	28.83 (5.79)
	Poor	26 (19.3%)	7.58 (2.58–19.05)	19.13 (6.11)

## Data Availability

The data presented in this study are available on request from the corresponding author.

## Supplementary Materials

The following are available online at https://www.mdpi.com/2072-6694/13/3/375/s1, Figure S1. Flowchart of the study with RCC patients. Figure S2. Levels and performances of plasma hPG_80_ in patients with RCC patients. A. hPG_80_ concentrations in RCC patients (*n* = 39) compared to 18–25 (*n* = 137) and 50–80 (*n* = 252) years old control groups. One-tailed Mann-Whitney test. B to C. Diagnostic discriminative accuracies of hPG_80_ in RCC patients compared to control groups aged 18–25 year old (B) or 50–80 year old (C) using Receiver Operating Characteristics (ROC) curve analysis. Table S1. Clinical and pathological characteristics for RCC patients. Table S2. hPG_80_ levels in RCC patients.
