# Supplementary material for: Prognostic Value of Plasma hPG80 (Circulating Progastrin) in Metastatic Renal Cell Carcinoma
Source: Cancers (Basel). 2021 Jan 20;13(3):375. doi: 10.3390/cancers13030375 (PMC7864155; doi:10.3390/cancers13030375)
Supplement: Supplementary file 1 [file cancers-13-00375-s001.pdf]

cancers

MDPI

Prognostic Value of Plasma hPG$_{80}$ (Circulating Progastrin) in Metastatic Renal Cell Carcinoma

Manish Kohli, Winston Tan, Bérengère Vire, Pierre Liaud, Mélina Blairvacq, Frederic Berthier, Daniel Rouison, George Garnier, Léa Payen, Thierry Cousin, Dominique Joubert, Alexandre Prieur

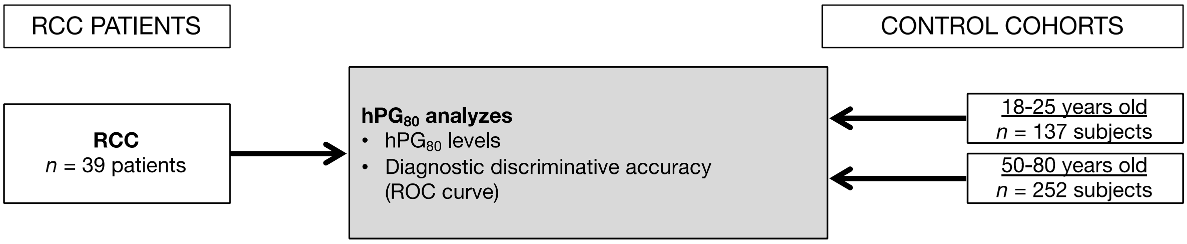

Figure S1. Flowchart of the study with RCC patients.

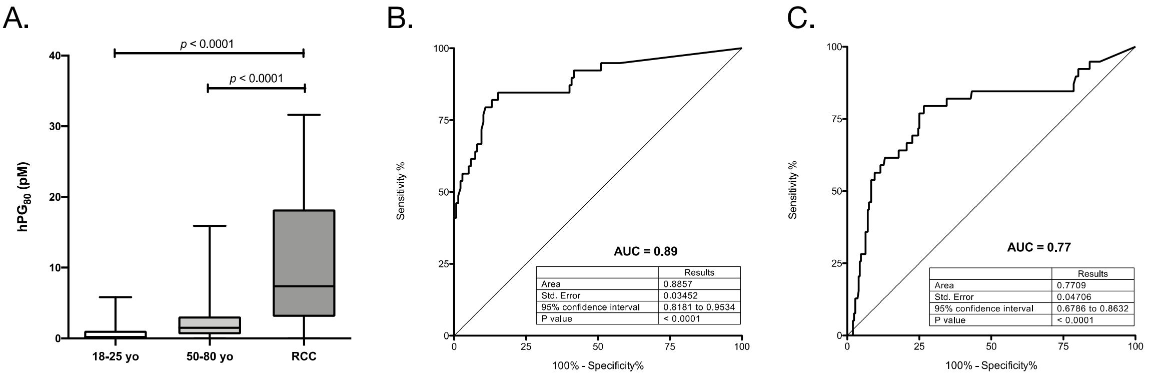

Figure S2. Levels and performances of plasma hPG$_{80}$ in patients with RCC patients. A. hPG$_{80}$ concentrations in RCC patients (n = 39) compared to 18-25 (n = 137) and 50-80 (n = 252) years old control groups. One-tailed Mann-Whitney test. B to C. Diagnostic discriminative accuracies of hPG$_{80}$ in RCC patients compared to control groups aged 18-25 year old (B) or 50-80 year old (C) using Receiver Operating Characteristics (ROC) curve analysis.

Table S1. Clinical and pathological characteristics for RCC patients.

		**RCC**
		N (%)
		n = 39
**Age, years**	Median (range)	67 (25–83)
**Gender**	Male	25 (64.1%)
	Female	14 (35.9%)
**Histology**	Clear cell	21
	Chromophobe	3
	Papillary	10
	Other	5
**Fuhrman Grade (G)**	G1	2
	G2	15
	G3	7
	G4	9
	Not applicable	5

	Unspecified	1
**Sarcomatoid differentiation**	Present	NA
	Absent	NA
**Necrosis**	Present	NA
	Absent	NA
**Clinical stage**	I	23
	II	4
	III	11
	IV	0
	Unspecified	1

NA: non available

Table S2. hPG_{80} levels in RCC patients.

		N	hPG80	
			Median (IQR), pM	Mean (SE), pM
**Patients**	RCC	39	7.36 (3.05-18.23)	10.73 (1.61)

Copyright: © 2021 by the authors. Licensee MDPI, Basel, Switzerland. This article is an open access article distributed under the terms and conditions of the Creative Commons Attribution (CC BY) license (http://creativecommons.org/licenses/by/4.0/).
